# Standardized terrestrial laser scanning dataset of forests: 3D point clouds and tree-level attributes from 121 plots across five European regions

**DOI:** 10.1016/j.dib.2026.113024

**Published:** 2026-06-24

**Authors:** Miriam Herrmann, Marius Derenthal, Ephraim Amos Schmidt-Riese, Marion Stellmes, Růžena Janoutová, Florian Arendholz, Daria Alison Bäte, Jonas Ernst, Arvin Fakhri, Piet Jaki, Florian Katerndahl, Fabian Kempfer, Chiara Mansi, Johann Meindl, Barbora Navrátilová, Florian Plewnia, Thomas Ruhtz, Marius Scholl, Asad Waseem, Fabian Fassnacht

**Affiliations:** a Freie Universität Berlin, Remote Sensing and Geoinformatics, Geographical Sciences, Malteserstr. 74-100, 12249, Berlin, Germany; b Technical University Berlin, Geoinformation in Environmental Planning Lab, Landscape Architecture and Environmental Planning, Straße des 17. Juni 145, 10623, Berlin, Germany; c Karlsruhe Institute of Technology (KIT), Geography and Geoecology, Reinhard-Baumeister-Platz 1, 76131, Karlsruhe, Germany; d Norwegian University of Life Sciences, Environmental Sciences and Natural Resource Management, P.O. Box 5003, 1432, ÅS, Norway; e Global Change Research Institute of the Czech Academy of Sciences, Bělidla 986/4a, 60300, Brno, Czech Republic; f University of Tuscia, Department of Ecological and Biological Sciences, Largo dell’Università s/n, 01100, Viterbo, Italy

**Keywords:** Forest structure, LiDAR, Picea abies, Pinus canariensis, Pinus sylvestris, Quercus spp., RIEGL, TLS

## Abstract

Forest structure strongly influences ecological processes, drives biodiversity patterns and habitat availability, and is directly linked to carbon storage. Terrestrial Laser Scanning (TLS) enables highly detailed three-dimensional characterization of forest ecosystems at the plot scale, capturing both canopy structure and understory vegetation with high spatial precision. However, many publicly available TLS datasets are limited in spatial extent or employ various acquisition protocols, complicating cross-site comparisons.

This article introduces a harmonized TLS dataset comprising 121 forest plots (50 m × 50 m each) sampled across five measurement campaigns in Germany, Czechia, Spain (Galicia and La Palma), and Norway. All campaigns used identical TLS acquisition protocols, the same scanner (RIEGL VZ-400i), and a fully standardized data-processing workflow to achieve maximum comparability. For each plot, the collection provides a registered and filtered 1 cm resolution 3D point cloud (.laz), along with comprehensive tree-level structural and compositional data (including diameter at breast height, tree height, and identity as deciduous or coniferous), standardized shapefiles (plot extent, scan positions, and tree positions), and a preview image for rapid visualization. Additionally, plot-level descriptors for terrain, climate, and soil are included.

The combination of a high number of plots, diverse environmental conditions, and strict methodological standardization enables robust comparative analyses of forest structural variability and cross-ecosystem comparisons of forest 3D structure. With additional reference data (e.g., labeling of the point clouds), the point clouds could be used for cross-site benchmarking of segmentation, inventory, and ecological modeling methods. It supports research on the relationships between forest structure and the environment.

## Graphical abstract

**Figure:**
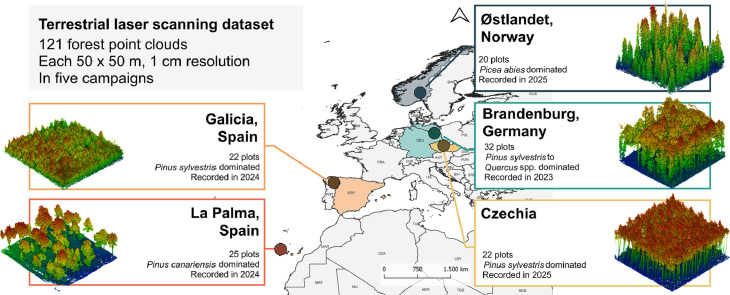

Specifications Table

**Table:** 

Subject	Earth & Environmental Sciences
Specific subject area	Standardized plot-based terrestrial laser scanning data for forest structure studies
Type of data	Point cloud (.laz); Shapefile (.shp); Table (.csv); Image (.png)
Data collection	TLS data acquired using a RIEGL VZ-400i, standardized scan design per 50 m × 50 m plot (target of 27 scan positions/plot, including tilted scans); acquisitions during vegetation peak; georeferenced using GNSS-RTK positioning. Co-registration, noise filtering, and subsampling (1 cm) were performed in RiSCAN PRO; tree metrics were extracted via the 3DFin plugin in CloudCompare.
Data source location	Brandenburg (Germany); La Palma (Canary Islands, Spain); Czechia (whole country); Galicia (Spain); Østlandet (Norway) For exact locations, see the provided shapefile in the data download.
Data accessibility	Repository Name: Zenodo Data identification number (DOIs): Master record: 10.5281/zenodo.18670608 [1] Campaign records: Brandenburg, Germany: 10.5281/zenodo.18670636 [2] La Palma, Canary Islands, Spain: 10.5281/zenodo.18847411 [3] Czechia: 10.5281/zenodo.18670739 [4] Galicia, Spain: 10.5281/zenodo.18846019 [5] Østlandet, Norway: 10.5281/zenodo.18847503 [6] Direct URL to data: Master record: https://zenodo.org/records/18670608 Campaign records: Brandenburg, Germany: https://zenodo.org/records/18670636 La Palma, Canary Islands, Spain: https://zenodo.org/records/18847411
	Czechia: https://zenodo.org/records/18670739 Galicia, Spain: https://zenodo.org/records/18846019 Østlandet, Norway: https://zenodo.org/records/18847503 Instructions for access: The main record contains aggregated metadata and plot location information. The Campaign records contain the full TLS data.
Related research article	None

## 1 Value of the Data

• This dataset provides a large, harmonized collection of terrestrial laser scanning (TLS) forest plot measurements acquired using a single instrument (RIEGL VZ-400i) and standardized scan protocols across five independent campaigns. The consistent acquisition settings, scan patterns, and processing workflows ensure that structural differences among plots primarily reflect ecological variability rather than sensor or acquisition effects and inconsistencies, enabling robust cross-site comparisons of forest structure.
• The dataset enables comparative analysis of forest structural properties, including tree density, basal area, canopy cover, tree height distribution, and stand density index, across climatically and structurally diverse forest systems. The combination of co-registered TLS point clouds, tree-level structural attributes, and terrain metrics extracted from the point clouds, together with environmental descriptors extracted from secondary datasets (e.g., climate and soil information), allows researchers to directly examine relationships between forest structure and environmental gradients. This supports interdisciplinary research in forest ecology and environmental remote sensing.
• The dataset provides detailed reference information on vertical forest structure and understory conditions. The high spatial resolution of the TLS measurements captures detailed three-dimensional information on lower canopy layers and understory vegetation that are often underrepresented in airborne laser scanning (ALS) datasets due to occlusion and lower point densities.
• In combination with auxiliary data or manual segmentations, the dataset can serve as a benchmark resource for the development and validation of automated algorithms for forest analysis from TLS data. The large number of consistently acquired TLS plots enables testing and training of both rule-based and machine-learning approaches for tasks such as tree detection, tree segmentation, canopy structure characterization, and the estimation of forest inventory metrics.
• The TLS measurements provide high-resolution reference data for scaling analyses that link detailed plot-level forest structure to observations from airborne and satellite platforms. Such data can be used to validate structural metrics derived from larger-scale remote sensing systems, to support upscaling approaches that connect detailed measurements with landscape- and regional-scale forest monitoring, and to analyze the effect of forest structure on remotely sensed signals.

## 2 Background

Recent advances in terrestrial laser scanning (TLS) have enabled the acquisition of detailed three-dimensional representations of forest structure, supporting applications in ecology and forestry research. However, differences in scanner hardware, plot design, sampling protocols, and data processing workflows have often limited the comparability of TLS datasets across sites and ecosystems.

To address this, we provide a harmonized dataset across five independent campaigns spanning Germany, Czechia, Spain (Galicia and La Palma), and Norway. All measurements were collected during the vegetation peak using the same instrument (RIEGL VZ-400i), the same scan protocol, and processed using a unified workflow. This dataset supports the assessment, calibration, and validation of forest inventory and remote sensing methods across different ecological contexts. This approach enabled the compilation of a plot-based dataset in which differences in forest structure are attributable to ecological differences rather than sensor or protocol variability, thus supporting robust comparative research, method development, and reproducibility.

## 3 Data Description

### 3.1 Data set structure

The main component of this dataset consists of 121 TLS forest plots. TLS data is accompanied by descriptive information in the form of shapefiles, tables, and preview images.

**Fig. 1:**
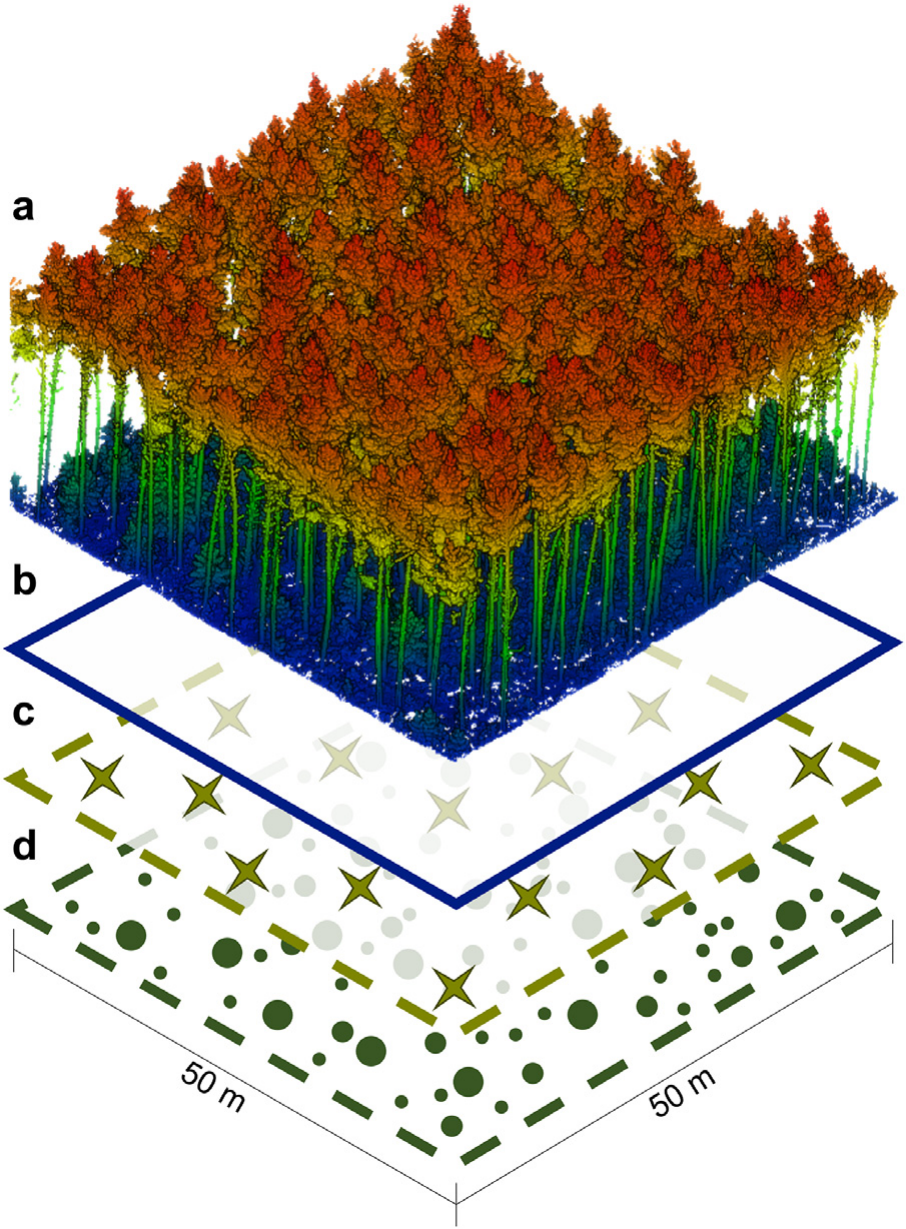
Spatial data provided for each plot includes the 3D point cloud (a), a shapefile of the plot extent (b), a shapefile of TLS scanning positions (c), and tree positions (d). Additionally, we provide an overview image (shown in a) and an overview table with information on terrain, forest structure, climate, and soil.

It is organized into a master record and five campaign-specific records (Table 1). The master record aggregates plot-level metadata and provides plot locations (Table 2), enabling users to filter and select campaigns for consecutive analysis. The campaign records contain the full TLS datasets, including point clouds, metrics describing plot forest structure, terrain, soil and climate, and ancillary files (Fig. 1; Table 3).

**Table 1:** Data set abbreviations, DOIs and sizes. The master record contains metadata for the 121 forest point clouds, and the campaign records contain the actual point cloud data.

Dataset	Abbreviation	DOI	Number of TLS forest plots	Complete size [GB]
Master record	-	10.5281/zenodo.18670608	(121)	0.01
Brandenburg, Germany	BB	10.5281/zenodo.18670636	32	44.61
La Palma, Spain	LP	10.5281/zenodo.18847411	25	26.31
Czechia	CZ	10.5281/zenodo.18670739	22	29.49
Galicia, Spain	ES	10.5281/zenodo.18846019	22	21.81
Østlandet, Norway	NO	10.5281/zenodo.18847503	20	40.78

**Table 2:** Content of the master record data set (doi:10.5281/zenodo.18670608).

Folder	Component	Description
Main folder	README.txt	Description of the structure and the components in the master dataset and the related campaign dataset
	overview_table.csv	Metadata on each recorded plot. Key variables include plot ID, point cloud file size, tree counts and attributes, terrain, climate, and soil descriptors
	README_overview_table.txt	Description of each column in the overview_table.csv
	plot_centers.shp	Shapefile of plot centers for all campaigns
	README_plot_centers.txt	Description of the plot_centers.shp and attributes in the shapefile

**Table 3:** Content of the campaign record data sets. The campaign abbreviation (Table 1) replaces XX in the component names.

Folder	Component	Description
Main folder	README.txt	Description of the structure and the components in the campaign dataset
	overview_table_XX.csv	Metadata on each recorded plot in this campaign, analogous to the overview_table.csv in the master record
	README_overview_table.txt	Description of each column in the overview_table_XX.csv
pointclouds	XX_01.laz, XX_02.laz, XX_03.laz, …	One point cloud (50 m × 50 m, 1 cm resolution) for each forest plot in the campaign
	README_pointclouds.txt	Description of the point clouds
preview_images	XX_01.png, XX_02.png, XX_03.png, …	Isometric visualization of the point cloud of each plot in the campaign as a preview image
	README_preview_images.txt	Description of the preview images
shapefiles	plot_areas_XX.shp	Shapefile with all plot areas in the campaign
	Scanpositions_from_RiScan_XX.shp	One shapefile with all single scan positions aligned to get the point cloud files
	README_shapefiles.txt	Description of the provided shapefiles and shapefile attribute tables
shapefiles/trees_per_plot	tree_positions_XX_01.shp, tree_positions_XX_02.shp, tree_positions_XX_03.shp, …	One shapefile for each plot with tree locations detected from the TLS point clouds and tree attributes (DBH, height, tree identity as deciduous or coniferous)

**Fig. 2:**
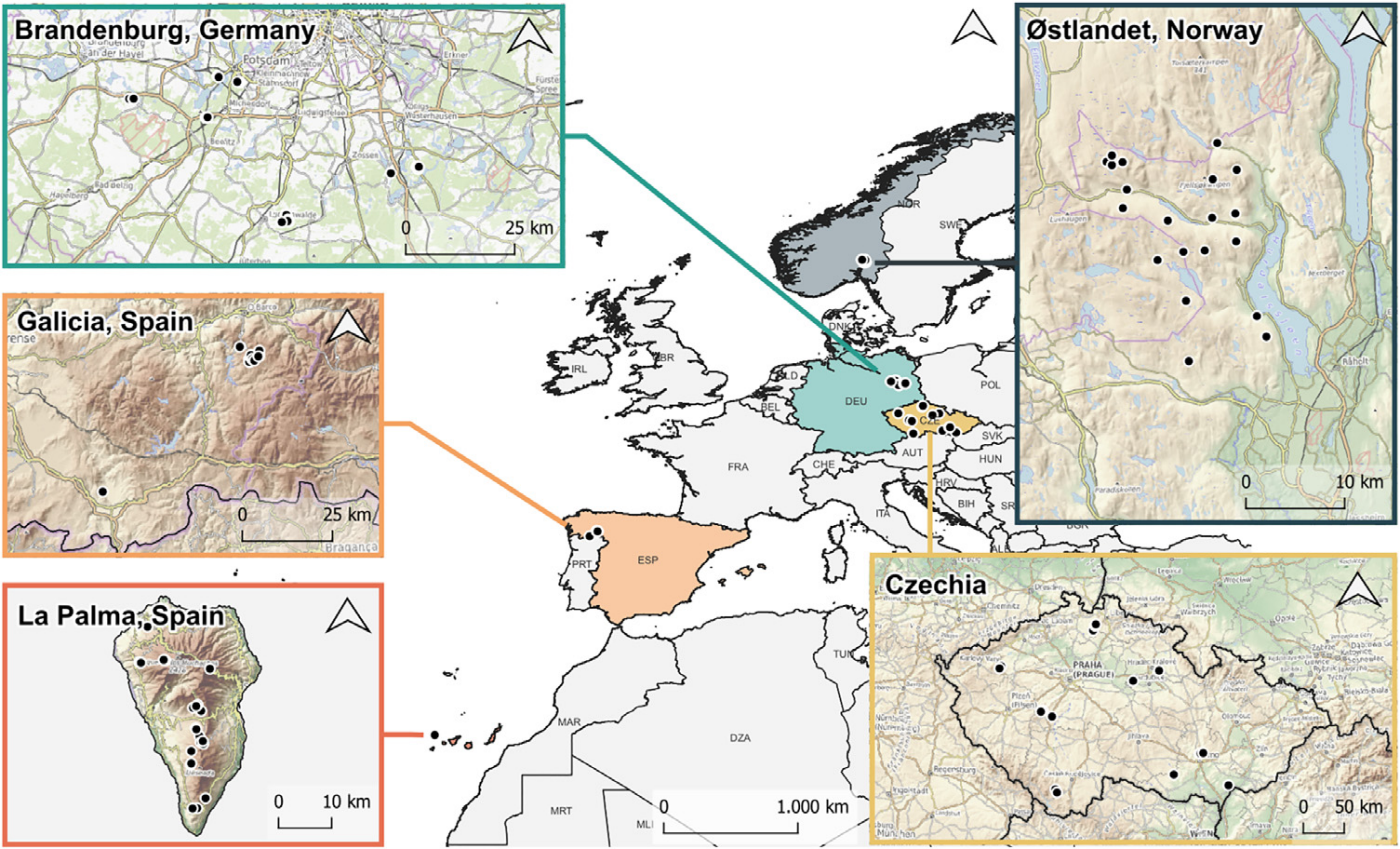
Overview map of the forest plots in the 5 different campaigns. Black dots: Plot locations. Background map in inset maps: OpenTopoMap.

To facilitate efficient data access and reduce download size when only a subset of plots is required, the dataset is distributed as separate records for the master dataset and individual campaigns (Fig. 2). Each record is provided as a single compressed (.zip) folder, with an internal structure as described in Table 2, Table 3.

### 3.2 Data components

#### 3.2.1 TLS point clouds (campaign records)

The 121 recorded TLS point clouds are sorted in campaign records and can be downloaded for each campaign separately under the corresponding DOI (Table 1).

For each forest plot, a co-registered, filtered, and subsampled three-dimensional point cloud file is provided (.laz-file with 1 cm spatial resolution). Each cloud covers a 50 m × 50 m area, with coordinates referenced to the local UTM zones (BB – EPSG 25,833, LP – 32,628, CZ – 25,833, ES – 25,829, NO – 25,832). Subsampling is performed to achieve an even point density of approximately 1 point per cm³, reducing storage requirements while maintaining the most relevant structural information.

Most point clouds contain on the order of 100 to 300 million points per plot, with file sizes usually between 1 and 2 GB per plot. Exact properties per plot are provided in the overview tables in the data download. Raw, unprocessed single scans are not shared due to their size but can be made available upon request.

#### 3.2.2 Preview images (campaign records)

For a quick overview, we provide one screenshot of each point cloud in an isometric view, colored according to height. These images are for visualization purposes only and not intended for quantitative analysis.

#### 3.2.3 Shapefiles (campaign records)

The 3D point cloud data in each campaign record is supplemented with 2D shapefiles containing additional information or information extracted from the point clouds, ready for analysis. The provided shapefiles show plot extents, scan positions, and tree positions. The shapefiles of each campaign are provided in the same coordinate reference system as the point cloud.

##### 3.2.3.1 Plot extent

Each campaign record includes one polygon shapefile containing the boundaries of all sampled plots (attributes: Table 4).

**Table 4:** Attributes given in the plot extent shapefile (plot_areas_XX.shp) for each campaign. Shapefiles are structured the same for each campaign, but are provided in a local coordinate reference system.

Column Name	Description	Unit / Type	Example Value
plot	Plot identifier. Combines campaign code and number	String	BB_01

##### 3.2.3.2 Scan position

This point shapefile records all scan positions used to generate the co-registered point cloud for each plot. Each point represents one TLS scan position; the exact scanner angle and height are given as attributes (Table 5).

**Table 5:** Attributes given in the scan positions shapefile (Scanpositions_from_RiScan_XX.shp) for each campaign. Shapefiles are structured the same across campaigns but are provided in local coordinate reference systems (BB – EPSG 25,833, CZ – 25,833, LP – 32,628, ES – 25,829, NO – 25,832)**.**

Column Name	Description	Unit / Type	Example Value
plot	Plot identifier. Combines campaign code and number	String	BB_01
scanpos	Scan position number within the plot	Integer	ScanPos001
Z	Scanner height above reference level	Float [m]	97.42
Roll	Scanner roll angle	Float [deg]	−3.191
Pitch	Scanner pitch angle	Float [deg]	−4.557
Yaw	Scanner yaw angle	Float [deg]	−152.146

##### 3.2.3.3 Tree positions

Tree-level information is provided in a separate point shapefile for each plot, with tree positions for all trees with a DBH ≥ 0.07 m. Trees were detected from the TLS point cloud using 3DFin [7]. The functional identity of each tree (deciduous or coniferous) was determined visually. The attributes given for each tree are directly derived from the point clouds and shown in Table 6.

**Table 6:** Attributes given in the tree positions shapefile (tree_positions_XX_01.shp) for each plot. Shapefiles are structured the same for each plot and campaign, but given in a local coordinate reference system.

Column Name	Description	Unit / Type	Example Value
tree_id	Unique tree identifier	Integer	01
height_m	Tree height (m), rounded to 2 decimals, derived from 3DFin [7]	Float [m]	20.53
DBH_m	DBH in meters, rounded to 2 decimals, derived from 3DFin [7]	Float [m]	0.34
identity	Visual classification (deciduous/coniferous)	String	deciduous

#### 3.2.4 Overview table (master record and campaign records)

For each plot, various metrics describing the data, data collection, forest structure, and ecological conditions are compiled (Table 7). Forest structure and small-scale terrain are extracted from the point cloud and summarized on the plot level. Climatic and soil information is extracted from global datasets for overview purposes.

**Table 7:** Columns given in the plot overview table for the master record and the campaign records. Each row in the overview table corresponds to one plot.

Column Name	Description	Type [Unit]	Example Value
campaign	Campaign abbreviation (only in the master record table)	String	BB
plot	Plot identifier. Combines campaign code and number	String	BB_01
**A – General information on the data**			
A_filesize_GB	Size of the point cloud file	Float [GB]	1.539
A_npoints	Number of points in the point cloud	Integer	126,285,919
A_crs_epsg	EPSG code of CRS	Integer	25833
A_extent_x_min	Minimum X (UTM) of plot extent	Float	369,249.76
A_extent_y_min	Minimum Y (UTM) of plot extent	Float	5803,924.6
A_extent_x_max	Maximum X (UTM) of plot extent	Float	369,299.75
A_extent_y_max	Maximum Y (UTM) of plot extent (The spatial extent is additionally provided as a polygon shapefile in the shapefiles folder.)	Float	5803,974.59
A_country	The country where the data were recorded	String	Germany
**B – Details on the data collection**			
B_aquisition_date_ YYYYMMDD	TLS acquisition date in YYYYMMDD format	Date [YYYYMMDD]	20230711
B_n_registered_singlescans	Number of registered single (raw) scans per plot	Integer	29
**C – Details on forest structure** Tree-level information is derived from TLS point clouds. Only trees with DBH ≥ 0.07 m are included.			
C_trees_n	Number of detected tree stems	Integer	71
C_coniferous_trees_n	Number of coniferous trees	Integer	7
C_deciduous_trees_n	Number of deciduous trees	Integer	64
C_coniferous_trees_percent	Percent coniferous trees	Float [%]	9.9
C_deciduous_trees_percent	Percent deciduous trees	Float [%]	90.1
C_dbh_mean_m	Mean DBH, calculated from DBH values derived using 3DFin	Float [m]	0.27
C_height_mean_m	Mean tree height, calculated from height values derived using 3DFin [7]	Float [m]	26.13
C_ba_m2_per_ha	Basal area per hectare, calculated from DBH values derived using 3DFin [7]	Float [m²/ha]	24.32
C_sdi	Stand density index, calculated from DBH values derived using 3DFin [7]	Float	444
C_canopy_cover_percent	Plot-level canopy cover, calculated from a 10 cm resolution canopy height model (CHM) derived from the point cloud. A raster cell is classified as canopy if vegetation height > 5 m	Float [%]	84.6
C_tree_species_main	Main tree species (genus or species, Latin name)	String	Quercus spp
C_tree_species_secondary	Secondary tree species (Latin name), ordered by occurrence in the plot from most to least common. If the stand is a pure stand, this column contains a “-"	String	Pinus sylvestris
**D – Terrain conditions** Terrain metrics are derived from a 1 m × 1 m resolution Digital Terrain Model (DTM) extracted from the TLS point cloud. The DTM is not included in the publication but is available upon request.			
D_median_slope_degrees	Median slope angle of all raster cells within the plot, representing the overall terrain steepness	Float [°]	6.3
D_slope_IQR_degrees	Interquartile range (75th percentile – 25th percentile) of slope values within the plot, describing the internal variability in terrain steepness	Float [°]	1.5
D_circular_mean_ aspect_degrees	Circular mean of aspect values within the plot (range 0–360°), values are measured clockwise from north and represent the dominant slope orientation	Float [°]	72
D_concentration_R	Concentration parameter (R) of aspect values, values near 1 indicate uniform slope direction, values near 0 indicate heterogeneous terrain or flat conditions	Float	0.97
D_elevation_range_m	The difference between the maximum and minimum elevation within the plot describes the vertical terrain relief	Float [m]	6.75
D_slope_class	Qualitative classification of terrain steepness based on median slope: • flat (≤ 5°) • gentle (5 - 15°] • moderate (15 - 30°] • steep (> 30°)	String	gentle
D_aspect_class	Qualitative cardinal direction classification based on median slope, circular mean aspect, and concentration R: • flat (mean slope ≤ 5°) • no dominant aspect (R ≤ 0.3) • north-facing (circular mean aspect 315–360 and 0 - 45°] • east-facing (45 - 35°] • south-facing (135 - 225°] • west-facing (225 - 315°]	String	east-facing
D_relief_class	Qualitative relief classification based on elevation range: • low relief (≤ 5 m) • moderate relief (5 - 15 m] • high relief (> 15 m)	String	moderate relief
**E – Climate classification** Climate information is extracted from global datasets and provided for overview purposes only [8,9].			
E_koeppen_geiger_short	Three-character Köppen-Geiger climate classification code, for interpretation, see the primary source [9]	String	Cfb
E_koeppen_geiger_long	Full Köppen-Geiger climate class classification name [9]	String	Temperate, no dry season, warm summer
E_annual_mean_temp_celsius	Mean annual temperature, derived from WorldClim data [8]	Float [ °C]	9.2
E_annual_precipitation_mm	Annual precipitation, derived from WorldClim data [8]	Integer [mm]	554
**F – Soil information** Soil information is based on the FAO/UNESCO Digital Soil Map of the World [10]. As it is extracted from a global dataset, it should serve only for overview purposes.			
F_dominant_soil_unit_short	Dominant FAO soil unit (short code) [10]	String	Bd
F_dominant_soil_unit_long	Full names of the dominant FAO soil unit [10]	String	Dystric Cambisols

An overview table for all plots is provided in the master record. This table can be used when filtering plots with specific characteristics. Additionally, a campaign-specific subset of the overview table is provided with each campaign, intended for users interested in a specific local point cloud set.

### 3.3 Campaigns

#### 3.3.1 Brandenburg, Germany

In the Brandenburg campaign in Germany, 32 forest plots were recorded. Field sampling took place from 11/07/2023 to 22/08/2023. Every plot covers a forest area of 50 m × 50 m (2500 m^2^), which is scanned with 25 to 42 single scans (median: 31.5). TLS data is stored in the .laz format with 1 cm spatial resolution in the coordinate reference system ETRS89 / UTM zone 33 N (EPSG 25833). The mean file size per point cloud is 1.4 GB, and the mean number of points per file is 123,066,142, corresponding to 49,226 points/m^2^.

The mean annual temperature in the plot locations is 9 °C, and the mean annual precipitation is 551 mm [8]. Plots are located in the temperate climate zone with no dry season and warm summers (Köppen Geiger climate classification: Cfb) [9]. The dominant soil types are Dystric Cambisols and Leptic Podzols [10].

The selection of plot locations in this campaign focused on covering a gradient from Pine (*Pinus sylvestris*) to Oak (*Quercus* spp.) dominated forests. Plot locations were selected based on species records from forest inventory data. All plots are located on state-owned land and are in active forestry for wood production.

The main tree species is *Pinus sylvestris* in 16 out of 32 plots, *Quercus* spp. in the other 16 plots. Half of the plots are pure stands, and the other half are mixed stands with the non-dominant species of the two species as a secondary species. The overall proportion of coniferous and deciduous trees in the dataset is fairly even, with 2254 scanned coniferous trees and 1646 deciduous trees. Forests are relatively dense with a mean basal area of 26.14 m^2^/ha and a mean canopy cover of 85%. The mean tree height is 24.1 m.

#### 3.3.2 La Palma, Canary islands, Spain

In the campaign on the Canary Island of La Palma, Spain, 25 forest plots were recorded. Field sampling took place from 01/03/2024 to 11/03/2024. Each plot covers a forest area of 50 m × 50 m (2500 m²), which is scanned with 19 to 34 single scans (median: 27). The lower number of scans in some plots is due to very easy forest conditions and high visibility throughout the plot. TLS data is stored in the .laz format with 1 cm spatial resolution in the coordinate reference system WGS84 / UTM zone 28 N (EPSG 32628). The mean file size per point cloud is 1.1 GB, and the mean number of points per file is 92,235,840, corresponding to 36,894 points/m^2^. The smaller number of points compared to the other campaigns is due to a less dense forest structure.

The mean annual temperature in the plot locations is 14.5 °C, and the mean annual precipitation is 438 mm [8]. 23 plots are located in the temperate climate zone with dry summers and warm winters, 2 plots are located in the arid and hot steppe climate zone (Köppen Geiger climate classification: Csb, BSh) [9]. The dominant soil unit in 20 plots is classified as Chromic Luvisols; five plots are classified as Lithosols [10].

Plots are located in pine forests, covering a gradient in tree and understory cover.

All plots are dominated by the canary island pine, *Pinus canariensis*. No secondary species were identified. All 2261 trees in the plots were classified as coniferous. The basal area and tree density are highly variable, ranging from 10.5 to 64.3 m²/ha, with a mean of 31.4 m^2^/ha. Canopy cover also ranges widely from 24% to 99% with a mean of 62%. The mean tree height is 18.4 m.

#### 3.3.3 Czechia

This campaign covers 22 plots of forested area across the Czech Republic. Field sampling took place from 01/07/2024 to 16/07/2024. Every plot covers a forest area of 50 m × 50 m (2500 m^2^), which is scanned with 27 to 30 single scans (median: 27.5). TLS data is stored in the .laz format with 1 cm spatial resolution in the coordinate reference system ETRS89 / UTM zone 33 N (EPSG 25833). The mean file size per point cloud is 1.3 GB, and the mean number of points per file is 115,576,081, corresponding to 46,230 points/m^2^.

The mean annual temperature in the plot locations is 7.9 °C, and the mean annual precipitation is 646 mm [8]. Plots are located in the cold climate zone with no dry season and warm summers (Köppen Geiger climate classification: Dfb) [9]. Due to the large area covered in the campaign, the dominant soil type also varies, with Dystric Cambisols in 8 plots, Gleyic Luvisols in 4 plots, and other soil types in smaller numbers of plots [10].

Plot locations were chosen to coincide with plots in the DendroNetwork (https://dendronet.cz/) [11]. The centres of 17 of the recorded plots match exactly those of the plots in the DendroNetwork infrastructure; 5 plots are placed close to plots in the infrastructure.

The main tree species is *Pinus sylvestris* (19 out of 22 plots). Common secondary species are *Quercus* spp. (present in 8 plots), *Larix decidua* (5 plots) and *Fagus sylvatica, Picea abies*, and *Tilia* spp. (3 plots each). The dataset is dominated by coniferous trees, most of which are *Pinus sylvestris*. 4314 trees in the dataset were identified as coniferous, 742 as deciduous. Forests are fairly dense with a mean basal area of 30.60 m^2^/ha and a mean canopy cover of 89%. The mean tree height is 22.7 m.

#### 3.3.4 Galicia, Spain

In the campaign in Galicia, Spain, 22 forest plots were recorded. Field sampling took place from 03/08/2024 to 17/08/2024. Every plot covers a forest area of 50 m × 50 m (2500 m^2^), which is scanned with 27 to 29 single scans (median: 27). TLS data is stored in the .laz format with 1 cm spatial resolution in the coordinate reference system ETRS89 / UTM zone 29 N (EPSG 25829). The mean file size per point cloud is 1.0 GB, and the mean number of points per file is 93,302,065, corresponding to 37,320 points/m^2^.

The mean annual temperature in the plot locations is 8.5 °C, and the mean annual precipitation is 1282 mm [8]. Plots are located in the temperate climate zone with dry and warm summers (Köppen Geiger climate classification: Csb) [9]. The dominant soil unit in the campaign area is Rankers [10].

All plots are located in intensively managed pine plantations with varying treatments (thinned, unthinned, pruned, unpruned). Age and species structure were comparably homogeneous, with less variance between the plots than in the other campaigns.

The main tree species in all plots is *Pinus sylvestris*. As a secondary species, *Quercus* spp. was found in two plots, whereas 20 plots were pine monocultures with no other tree species. The dataset is thus highly dominated by coniferous trees. 3556 trees in the dataset are coniferous, 23 are deciduous. The mean basal area of the stands is 21.23 m^2^/ha, the mean canopy cover 72%. Compared to the other campaigns, the trees are young and small, with a mean tree height of 12.6 m.

#### 3.3.5 Østlandet, Norway

During the campaign in Østlandet, Norway, 20 forest plots were recorded. Field sampling took place from 13/08/2025 to 23/08/2025. Every plot covers a forest area of 50 m × 50 m (2500 m^2^), which is scanned with 20 to 51 single scans (median: 40). The low number of scans taken in some plots is due to steep terrain and thus dangerous scanning conditions. TLS data is stored in the .laz format at 1 cm spatial resolution in the coordinate reference system ETRS89 / UTM zone 32 N (EPSG 25832). The mean file size per point cloud is 2.1 GB, and the mean number of points per file is 155,673,982, corresponding to 64,692 points/m^2^.

The mean annual temperature in the plot locations is 3.0 °C, and the mean annual precipitation is 827 mm [8]. 17 plots are located in the cold climate zone with no dry season and cold summers (Köppen Geiger climate classification: Dfc) [9]. Three plots are located in the cold climate zone with no dry season and warm summers (Köppen Geiger climate classification: Dfb) [9]. The dominant soil unit for all plots is Orthic Podzols [10].

The 20 selected plots are a subset of the BioDivAbove project (https://www.nmbu.no/en/research/projects/biodiversity-mapping-forests-above-biodivabove), which seeks to map the biodiversity of spruce-dominated boreal forests. Plots depict gradients in forest age, productivity, and topographic wetness. Forest management is, in most plots, less intensive than in the other campaigns, as evidenced by more dead wood, a less even-aged tree structure, and highly variable stand structure between plots.

The main tree species in all plots is *Picea abies*. 8 plots are pure *Picea abies* stands, in 12 plots, *Betula* spp. is present as a secondary species. Overall, the dataset is dominated by coniferous trees: 5619 of the scanned trees are coniferous, and 303 are deciduous. Basal area and canopy cover vary widely (Basal area: 14.98 to 57.96 m^2^/ha, mean: 29.31 m^2^/ha; canopy cover: 31% to 94%, mean: 67%). The mean tree height is 17.0 m.

## 4 Experimental Design, Materials and Methods

### 4.1 Plot locations

All plot locations were chosen before the start of each campaign to avoid sampling bias introduced by data-acquisition teams selecting plots based on field-observed forest structure.

For the campaigns in Brandenburg (Germany), La Palma (Spain), and Galicia (Spain), plot locations were selected using a stratified random sampling approach.

For Brandenburg, Germany, the constraining factors were that plots needed to be placed on state-owned forest and to cover a gradient from pine (Pinus sylvestris) to oak (Quercus spp.) dominated. Species data for the stratification were obtained from the forest inventory data of the federal state of Brandenburg (Forstliche Grundkarte Brandenburg).

In La Palma, Canary Islands, Spain, plot locations were selected by accessibility (close to roads or trails visible in Open Streep Map data and no steep slope), to be located in pine forests, and to cover a range of tree cover situations (low, middle, and high) and understory cover (low, middle, and high) classes, which were visually assessed from Google earth and Google street view images [12].

In Galicia, Spain, plot locations were placed within a research area designed to assess different forest management treatments on the growth of *Pinus sylvestris* for commercial use, managed by AMETLAM, a company specializing in forest management (https://ametlam.gal/en/projects/). Plot locations were selected based on their accessibility, with a minimum distance to asphalted roads of approximately 50 m, and to represent a range of stand age classes. The age structure was approximated prior to fieldwork using visual interpretation of high-resolution imagery available on Google Earth [12].

In the potential plot area, random points representing the plot centers were placed using QGIS [13].

For the campaigns in Czechia and Østlandet, Norway, plot locations were placed to coincide with plots from other research projects.

In Czechia, plots were selected from the DendroNetwork (dendronet.cz) infrastructure based on pine (*Pinus sylvestris*) dominance. Species data used for this selection were collected during infrastructure field inventories. Additional plots were placed randomly but close to existing plot locations.

In the Østlandet, Norway, campaign plots were chosen as a subset of the BioDivAbove project (https://www.nmbu.no/en/research/projects/biodiversity-mapping-forests-above-biodivabove) to cover a gradient of forest age, productivity, and topographic wetness. Norway spruce (*Picea abies*) is the dominant tree species in the plots and economically the most important species in the area. The plots are situated on the property of Mathiesen Eidsvold Værk, a private forest company.

### 4.2 Plot setup

**Fig. 3:**
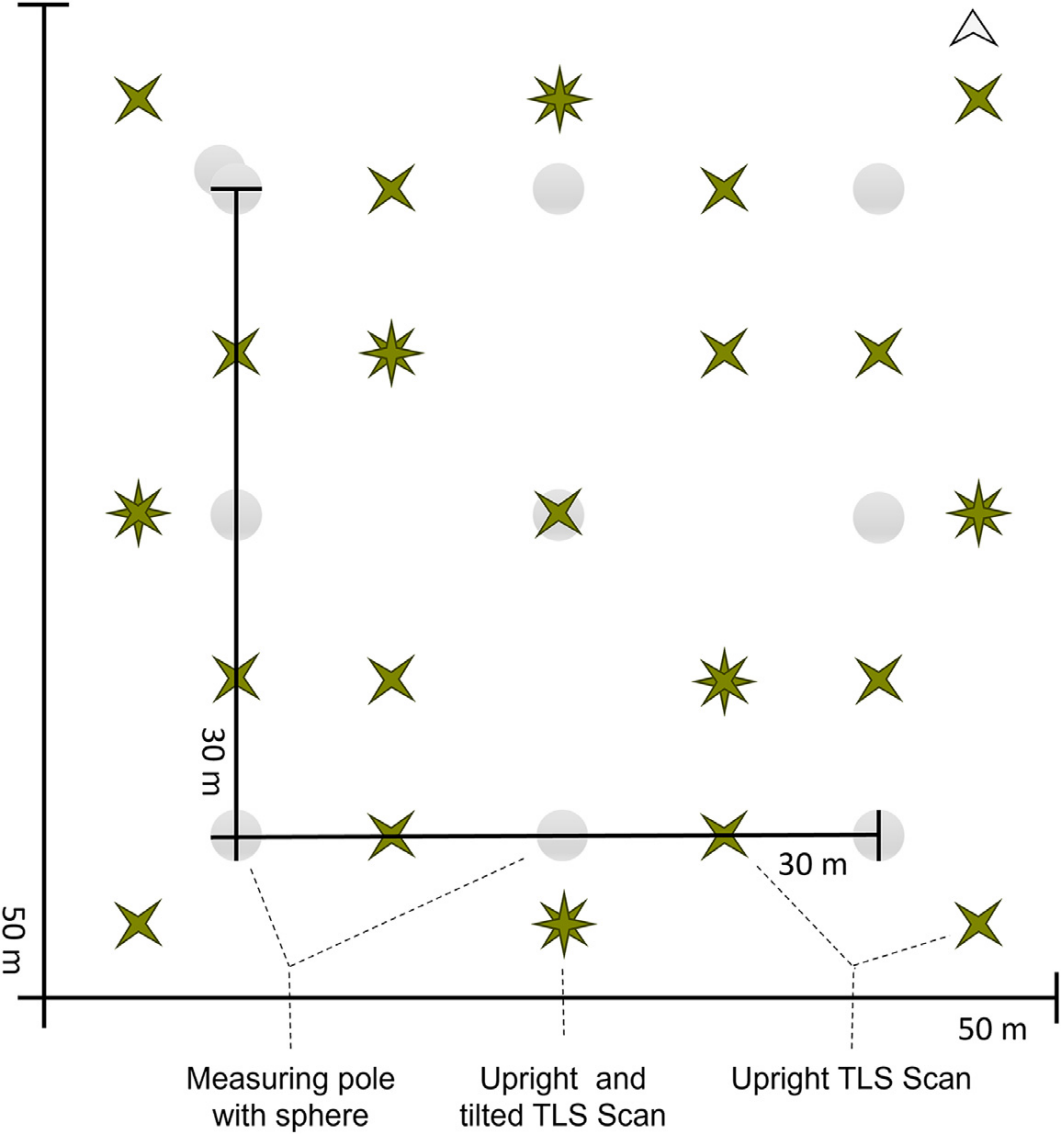
Scanning pattern used for all Terrestrial Laser Scanning data acquisitions. First, a 30 m × 30 m plot was established using a tape measure and a compass. Plot corners, the middle of each side, and the center were marked using poles and spheres (grey circles). Second, the plot was scanned with the RIEGLl VZ-400i, starting in the plot center. Point clouds were later cropped to 50 m × 50 m.

The plot setup was consistent across all campaigns. First, a handheld GPS device was used to locate the plot center. Second, a compass and measuring tape were used to establish a 30 m × 30 m plot around the plot center. The plots’ sites were oriented toward the cardinal directions. Plot corners, edge midpoints, and the center point were marked using measuring poles and polystyrene spheres (Fig. 3). To facilitate orientation during the scanning process, the pole in the north-west corner of the plot was marked using a double sphere.

### 4.3 TLS data acquisition

All plots were scanned using a RIEGL VZ-400i terrestrial laser scanner (RIEGL, Horn, Austria), operated with an angular resolution of 0.04°, which corresponds to a resolution of 7 mm at a distance of 10 m to the scanner, a pulse repetition frequency of 1200 kHz, a maximum range of 250 m, and a field of view of 100° vertical × 360° horizontal.

Each plot was scanned to approximate a previously defined pattern with 27 positions, using both level and tilted scans to maximize canopy and stem coverage (Fig. 3). In dense stands, additional scans were included to reduce occlusions. Scanning began at the plot center and moved outward in a spiral. This order of single scans taken improved the alignment of scan positions, as a dense cloud in the middle of the plot formed first, which is visible in many of the other scan positions. The standardized pattern was occasionally augmented or adapted on site to accommodate local forest structure or terrain. If scanning conditions were dangerous due to steep terrain, the scanning pattern was not strictly followed. Please refer to the provided shapefile for the actually used scan positions (x, y, and z device orientation) in each plot.

During all data acquisitions, one member of a two-person team (MH or MD) was present to ensure the established protocol was followed and to maintain comparability between campaigns.

### 4.4 TLS data processing

Individual scans were processed in RiSCAN PRO to retrieve point clouds. Used versions were 2.19.3 for the first 4 campaigns and 2.23 for the campaign in Østlandet, Norway.

First, single scans were filtered for noise, excluding points with reflectance values below −23.8 dB. This value was chosen empirically because it provided a good balance between excluding misplaced hits and minimizing the loss of visually plausible points. Misplaced hits mainly resulted from objects being too close to the scanner, e.g., the ground surface in tilted scans.

**Fig. 4:**
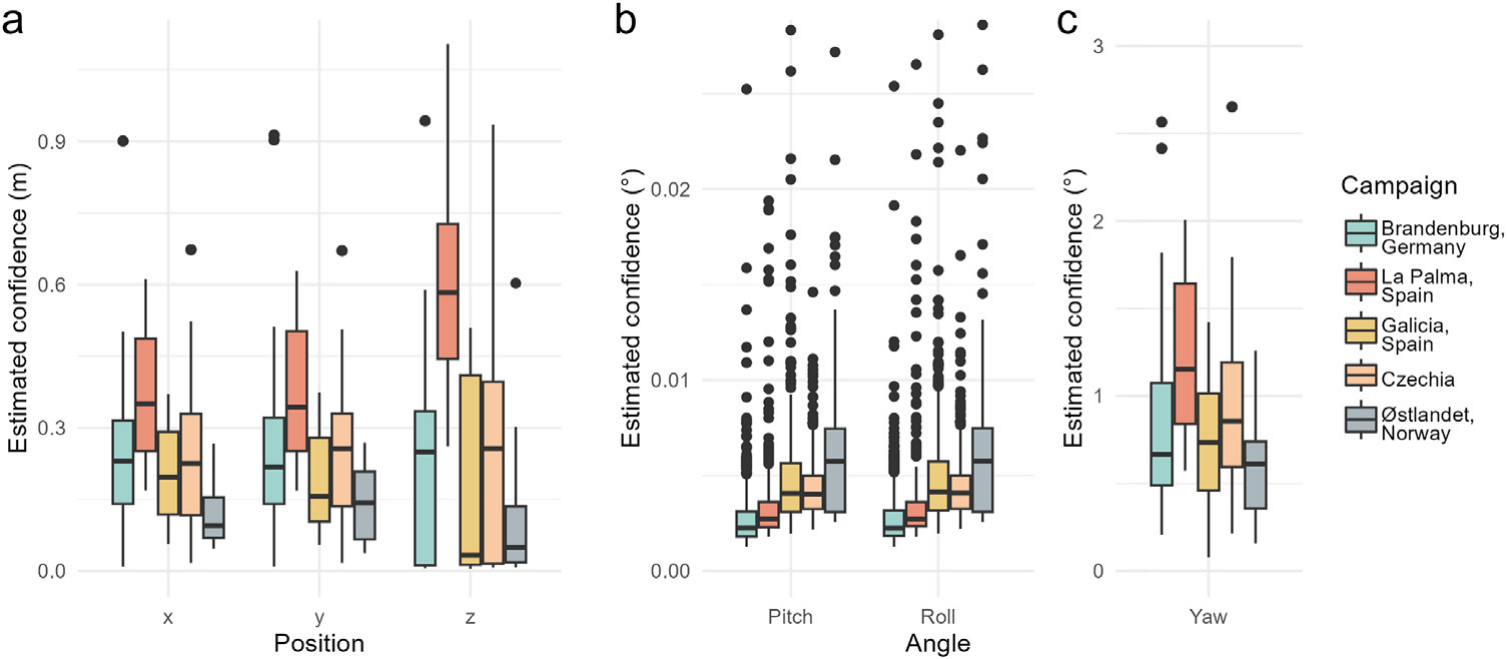
Internally estimated confidence for the position of single scans.

Second, single scans were registered using the settings for outdoor, non-urban environments. The final confidence in the accuracy of single-scan positions was internally assessed using RiSCAN PRO. Across all campaigns, the mean position confidence was 29 cm in the x direction, 30 cm in the y direction, and 34 cm in the z direction. The mean confidence of roll, pitch, and yaw was 0.004, 0.005, and 0.7°, respectively. Campaign-wise values are shown in Fig. 4. Position estimates for the La Palma, Spain, campaign are worse than for other campaigns, as RTK positioning services were not available to us during this campaign.

We, however, did not rely on the internal quality assessment measurements to determine the quality of scan alignments, as we found them arbitrary for some of the scanned forest ecosystems. In RiSCAN PRO, registration quality is usually internally assessed using residual distances between planar patches identified across individual scan positions. These plane distances are reported as a quality assessment metric. We observed that in dense forest ecosystems, this metric did not consistently reflect the scans' actual alignment quality. In several cases, the automatic registration and subsequent plane-matching distances indicated satisfactory alignment despite visible mismatches between scans, whereas in other cases, no valid solution was obtained through automatic alignment using this measure. These issues occurred particularly in plots with dense herb-layer vegetation, small-diameter trees, or trees with many low branches, where distinct planar surfaces on the ground or stems were limited. Therefore, we decided to use a visual, more subjective quality check of scan alignment.

After registration, the alignment of all scan positions was visually checked. For that, vertical and horizontal transects were cut through the point cloud. Alignment for scan positions that were not well aligned with the bulk of the scans (visible surfaces 3 cm or more apart) was removed. Alignment was repeated for those positions until a visually satisfactory result was achieved. Scanning data of positions that could not be aligned to the rest of the cloud was removed.After successful registration, single scans were merged, and the complete point cloud of each plot was subsampled to 1 point per 1 cm^3^ (uniform spatial resolution of 1 cm), ensuring consistent density across all plots for quantitative analysis. This step was applied to balance file size and spatial detail.

Lastly, plot clouds were cropped using the lidR package in R [[14], [15], [16]] to the 50 m × 50 m plot extent. This plot is a 10 m buffer around the 30 m × 30 m plots established in the field. The larger size was chosen because even the margin of point clouds is well covered, given the large number of scans in the scanning pattern.

All plots were processed by the same person (MH) to ensure comparability of the visual quality checks performed during processing. Unprocessed individual scans are not included in the shared dataset due to data size constraints, but can be made available upon request.

### 4.5 Tree detection and classification

Tree stem detection and tree attribute extraction were performed for each cloud using the 3DFin plugin in CloudCompare (version 2.13 beta), with default settings [7,17]. Trees with a DBH < 0.07 m were removed from the dataset, as these smaller stems belonged to shrubbery or small understory trees, which are not the focus of the dataset. For each detected tree, DBH and height were extracted from the point cloud using 3DFin [7]. Extracted measurements were not validated using field measurements, but analyses in comparable forest ecosystems with comparable instruments suggest good agreement between field measurements and extracted values [7]. Results tables from 3DFin were transferred to shapefiles containing tree position, DBH, and height using the sf package in R [16,18]. For each detected tree, a tree functional identity as deciduous or coniferous was assigned based on the visual appearance of the tree's shape in the point cloud using CloudCompare (version 2.13 beta) [17]. Visual comparison of the point cloud data with the detected tree positions showed good agreement. During the assignment of the function identity, during which each tree was visually assessed in the point cloud and checked against the detected positions, we did not find any errors in the identified tree positions. All plots were processed by the same person (MH) to ensure the comparability of the visual assignments.

### 4.6 Compiling of metadata

For overview purposes, various properties of the point cloud and the depicted forest plots, as well as additional data from global climate and soil datasets, were compiled in an overview table using the sf, terra, and lidR packages in R [16,18,19].

#### 4.6.1 Forest structure

Most forest structure parameters were extracted from the tree positions shapefile (chapter 4.5). Additionally, a canopy height model (CHM) was generated for each plot, and crown cover was calculated as a percentage. For this, the point cloud was height-normalized using points classified as ground (chapter 4.6.2), and a canopy height model was created using the rasterize_canopy function from the lidR package in R [[14], [15], [16]]. Each raster cell with a height above 5 m was counted as canopy.

All extracted metrics are listed and described in Table 7 under C – Details on forest structure.

#### 4.6.2 Terrain information

A ground classification was performed for each point cloud using the cloth simulation filter with the lidR package in R [[14], [15], [16],^20^]. The cloth resolution was set to 0.5 m, and a class threshold of 0.05 m, and a rigidness of 3 were applied. From the ground points, a digital terrain model with 1 m × 1 m resolution was created using Delaunay Triangulation.

From these digital terrain models, information on the local terrain conditions on the plot level was extracted. All extracted metrics are listed and described in Table 7 under D – Terrain conditions.

#### 4.6.3 Climate and soil

Descriptors of climate and soil conditions were obtained by overlaying plot locations with corresponding global raster datasets [[8], [9], [10]]. For climate, we extracted annual precipitation and mean temperature from Worldclim 2, a dataset that interpolates MODIS climate data at 1 km^2^ resolution [8], and a climate classification from Köppen-Geiger maps, also at 1 km^2^ resolution [9]. For soil, we extracted the dominant soil type from the FAO digital soil map of the world [10]. The exact extracted metrics are listed and described in Table 7 under E – Climate classification and F – Soil information. Details on the data can be found in the provided primary sources.

The global datasets were chosen instead of local ones to gain comparable information over all campaigns. Because those data sets have a much coarser resolution than the other metadata, which is extracted directly from the point clouds, they should be used for overview purposes only.

## Limitations

• The dataset represents a temporal snapshot of forest structure. Each plot was scanned only once, preventing analyses of temporal dynamics such as growth, mortality, or structural change.
• Detailed plot-level information on forest management history and silvicultural treatments is generally not available. Short descriptions of typical forestry practices were added for each campaign region to provide contextual information, but management data cannot be incorporated directly into plot-level analyses.
• Tree species are classified only into two functional groups: coniferous and deciduous. Species information at the individual tree level is not included.
• All scans were acquired during leaf-on conditions. This provides realistic canopy and foliage representation, but the presence of leaves may also have increased occlusion of stems, ground surfaces, and understory vegetation.
• Despite standardized scanning protocols and the use of additional tilted scans, some occlusion of structural elements may persist, particularly in dense stands. Minor residual co-registration errors between scans may remain.
• Environmental variables were extracted from global datasets and represent regional conditions rather than plot-level measurements. Structural metrics derived from the point clouds, like DBH measurements, lack independent ground-truth validation data.

## Ethics Statement

The authors have read and followed the ethical requirements for publication in Data in Brief and confirm that the current work does not involve human subjects, animal experiments, or any data collected from social media platforms.

## Credit Author Statement

**Miriam Herrmann**: Conceptualization, Methodology, Data collection, Data Curation, Writing - Original Draft, Writing - Review & Editing. **Marius Derenthal, Ephraim Schmidt-Riese, Marion Stellmes, Růžena Janoutová**: Data collection, Writing - Review & Editing. **Florian Arendholz, Daria Alison Bäte, Jonas Ernst, Arvin Fakhri, Piet Jaki, Florian Katerndahl, Fabian Kempfer, Chiara Mansi, Johann Meindl, Barbora Navrátilová, Florian Plewnia, Thomas Ruhtz, Marius Scholl, Asad Waseem**: Data collection. **Fabian Fassnacht**: Supervision, Conceptualization, Data collection, Writing - Review & Editing.

## Data Availability

ZenodoHarmonized terrestrial laser scanning forest dataset: 3D point clouds and tree-level attributes from five campaigns in Europe (Original data). ZenodoTerrestrial laser scanning forest dataset from Østlandet, Norway: 20 TLS point clouds (50 m x 50 m, 1 cm resolution) (Original data). ZenodoTerrestrial laser scanning forest dataset from Czechia: 22 TLS point clouds (50 m x 50 m, 1 cm resolution) (Original data). ZenodoTerrestrial laser scanning forest dataset from Brandenburg, Germany: 32 TLS point clouds (50 m x 50 m, 1 cm resolution) (Original data). ZenodoTerrestrial laser scanning forest dataset from La Palma, Spain: 25 TLS point clouds (50 m x 50 m, 1 cm resolution) (Original data). ZenodoTerrestrial laser scanning forest dataset from Galicia, Spain: 22 TLS point clouds (50 m x 50 m, 1 cm resolution) (Original data).
